# Bacteriological spectrum, extended-spectrum β-lactamase production and antimicrobial resistance pattern among patients with bloodstream infection in Addis Ababa

**DOI:** 10.1038/s41598-023-29337-x

**Published:** 2023-02-06

**Authors:** Adane Bitew, Amanuel Adane, Abera Abdeta

**Affiliations:** 1 Department of Medical Laboratory Science, College Health Sciences, Addis Ababa University, Addis Ababa, Ethiopia; 2 Saint Peter’s Specialized Tuberculosis Referral Hospital, Addis Ababa Administrative Region, Addis Ababa, Ethiopia; 3 National Clinical Bacteriology and Mycology Reference Laboratory, Ethiopian Public Health Institute, Addis Ababa, Ethiopia

**Keywords:** Microbiology, Health care, Medical research

## Abstract

Bloodstream infection coupled with drug resistance in bloodborne bacteria is a major health problem globally. The current study sought to identify the bacterial spectrum, extended-spectrum -lactamase production, and antimicrobial resistance pattern in patients with bloodstream infection. This prospective cross-sectional study was conducted at Arsho Advanced Medical Laboratory, Addis Ababa, Ethiopia from January 2019- until July 2020. Blood collected from patients was inoculated into blood culture bottles and incubated appropriately. Identification, antimicrobial susceptibility testing, and extended-spectrum β-lactamase-production were determined with the VITEK 2 compact system. Of the samples collected, 156 (18.5%) were culture-positive. *Klebsiella pneumoniae and Staphylococcus epidermidis* were the dominant isolates. In Gram-negative bacteria, the prevalence of drug resistance was the highest against ampicillin (80.8%) and the lowest against imipenem (5.2%). While in Gram-positive bacteria it was the highest against clindamycin and the lowest against vancomycin and daptomycin. The prevalence of multi-drug resistance and extended-spectrum β-lactamase production of Gram-negative bacteria were 41.6% and 34.2%, respectively. The prevalence of bloodstream infection was 18.5%. Serious life-threatening pathogens including *S*. *aureus, Klebsiella pneumoniae, Acinetobacter baumannii, Pseudomonas aeruginosa, Escherichia coli*, and *Enterobacter spp* was predominant. The prevalence of multi-drug resistance to both Gram-positive and Gram-negative bacteria and extended-spectrum β-lactamase-production were high but prevalence of carbapenem resistance was low. All these situations call for the establishment of strong infection control strategies, a drug regulatory system, and established antibiotic stewardship in healthcare settings.

## Introduction

Bloodstream infection is among the most common hospital and community-acquired infections, causing substantial death and morbidity globally^1,2^_._ The infection accounts for 10–20% of all hospital-acquired infections and ranks 8th in causing death^3^. About 48.9 million cases of bloodstream infection (BSI) and 11.0 million BSI-related deaths were reported in 2017^4^. In sub-Saharan countries, BSI is mostly seen in children below 5 years of age, and the death rate of children in developing countries versus the developed ones is found to be in the range of 100–250 and 10–30 per 1000, respectively^4^.

The rapid increase and spread of antibiotic resistance at different levels in the community and device-associated healthcare infections have become one of the three most important problems facing human health^5–7^. The co-existence of a high burden of infectious diseases and the rapid increase and spread of antimicrobial resistance have made the problem more serious in developing countries^8^. Consequently, antimicrobial resistance is estimated to cause 700,000 deaths per year, a number projected to rise to 10 million deaths annually by 2050^9^.

A wide range of Gram-negative and Gram-positive bacteria have been isolated in BSIs, among which *Acinetobacter spp*, *P. aeruginosa*, *E. coli,* and *K. pneumoniae* are the predominant Gram-negative bacteria, while coagulase-negative staphylococci (CoNS), *S. aureus*, enterococci, and alpha-hemolytic streptococci are the most common Gram-positive bacteria. However, the predominant bacterial species in BSI differ from setting to setting as the result of global differences in epidemiological and geographic features across regions^10^ These bacteria have also been recognized as the most serious multi-drug-resistant pathogens^11^. The situation is more serious in low-income countries, where drug abuse is a common problem^12^. Bacterial isolates that are non-susceptible to at least one drug in three or more drug categories are multi-drug resistance. Limiting uptake of a drug, modification of a drug target, inactivation of a drug, and active efflux of a drug are the main mechanisms of bacterial drug resistance.

Shortage of efficient diagnostic microbiology laboratories and difficulty in accessing effective antibiotic therapy for resistant pathogens have also remained major problems in low-income countries^8,13,14^. In low-income countries, identification and drug susceptibility testing of bacterial pathogens have been performed by a routine procedure involving a few biochemical tests and an agar diffusion assay against a few antibacterial agents, both of which are less accurate. Against this backdrop, the current study was designed to use the VITEK 2 compact system to determine the prevalence of BSI, the spectrum of bacterial bloodstream pathogens, the prevalence of extended-spectrum β-lactamase-producing Gram-negative bacteria, and their antimicrobial resistance profile.

## Materials and methods

### Study setting, design, and population

This prospective cross-sectional study was conducted at Arsho Advanced Medical Laboratory, Addis Ababa, Ethiopia, from January 2019 until July 2020. Patients suspected of having a bloodstream infection and those who fulfilled the standard operating procedure (SOP) of blood sample collection in the laboratory were included in the study.

### Specimen collection and inoculation

Before blood collection, the skin of each study patient was disinfected with 70% alcohol and subsequently with povidone-iodine. About 10 or 5 ml of venous blood in duplicate sterile tubes were collected aseptically from adults and children, respectively, by qualified nurses. Blood samples were inoculated into duplicate blood culture bottles containing 50 mL (adults) and 25 mL (for children) of sterile brain–heart infusion broth (Oxoid, Basingstoke, Hampshire, UK) under a biosafety cabinet. Blood culture bottles that showed signs of bacterial growth were subjected to Gram staining followed by sub-culturing into a blood agar base (Oxoid, Basingstoke, Hampshire, UK) to which 10% sheep blood was added, Chocolate agar (Oxoid, Basingstoke, Hampshire, UK**),** and MacConkey agar (Oxoid, Basingstoke, Hampshire, UK) after overnight, 48, and 72 h of incubation at 37 °C. Blood agar and chocolate agar plates were incubated at 37 °C in a 5% CO_2_ incubator for 24–72 h, while MacConkey agar plates were kept at 37 °C aerobically for 24 h. A terminal subculturing was done on chocolate agar for bottles that did not show visible growth within 7 days before being reported as negative. Blood culture was considered positive if growth was detected in both duplicate blood culture bottles only.

### Identification, antimicrobial susceptibility testing, and production of Extended Spectrum β- Lactamase

Identification, antimicrobial susceptibility testing, and production of Extended Spectrum β- Lactamase were determined with the automated VITEK 2 compact system (bioMérieux, France) as per the instruction of the manufacturer.

### Test for carbapenemase production

Test for carbapenemase production of bacterial isolates which were resistant to imipenem (IPM 10 µg) and, meropenem (MEM 10 µg) and ertapenem (ERT 10 µg) were subjected for confirmation for carbapenemase production. Confirmation for carbapenemase production in Gram-negative bacteria was conducted by Modified Hodge Test (MHT) where Mueller–Hinton agar plate was inoculated with a 1:10 dilution of a 0.5 densitometer standardized suspension of over-night sub-cultured *E. coli* ATCC 25922 and streaked for confluent growth using a swab. A 10 µg ertapenem disk was placed in the center, and each test isolate was streaked from the disk to the edge of the plate. A positive Modified Hodge Test (MHT) was indicated by clover leaf-like indentation of the *E. coli* ATCC 25922 growing along the test organism growth streak within the disk diffusion zone.

### Data quality assurance and quality control

Pre-analytica**l** and analytical procedures were performed following the standard operational procedure (SOP) of Arsho Advanced Medical Laboratory. The extracted information (post-analytical activities) such as laboratory findings were checked for eligibility, completeness, and consistency and recorded before entry into a statistical tool.

### Data analysis and interpretation

The data was collected, cleaned and analyzed using SPSS version 20. Frequency and percentages of MDR, carbapenemase and ESBL producing gram-negative bacteria were calculated. Tables were used for data presentation.

### Ethics statement and patient consent

The present work was conducted in accordance with the Declaration of Helsinki. All ethical considerations and obligations were duly addressed, and the study was carried out after obtaining ethical clearance from the ethical committee of the institute of the Advanced Medical Laboratory, Addis Ababa, Ethiopia. Informed consent was obtained from all subjects and/or their legal guardian(s). Personal information of patients and guardians was treated confidentially. Briefly, the aim of the work and its benefits were clearly described by each adult patient and the guardian. Adult patients and their guardians were not obliged to be involved in the study if they did not agree to participate in it. Once they consented to participate in the study and became disinterested to continue, they were free to withdraw from the study at any time during the course of the study.

## Results

### Socio-demographic characteristics

The demographic characteristics of the study patients are shown in Table 1. A total of 844 patients were included in the current work. Among these, 485 (57.5%) were females, and 359 (42.5%) were males. The majority of the study patients were in the age group of 25–44, and the least were in the age group of < 1 year (40; 4.7%). Out of the 844 blood samples collected, 156 (18.5) were culture-positive. Regarding gender, of 485 female patients, 88 (10.4%) were culture-positive. Similarly, of 359 male patients, 68 (8.1%) were culture-positive. Females were more affected than males. Regarding age, patients in the age group of 25–44 (5.1%) were more affected, followed by the age group 45–64 (4.7%), and by the age group > 65 (2.8).

**Table 1:** Demographic characteristics and bloodstream infection per different categories of patients (844).

Variables	Category	Number of blood samples per category (n, %)	Culture negative per category (n, %)	Culture positive per category (n, %)
Sex	Female	485 (57.5)	397 (47.0)	88 (10.4)
	Male	359 (42.5)	291 (34.5)	68 (8.1)
	Total	844 (100)	688 (81.5)	156 (18.5)
Age group	< 1	40 (4.7)	20 (2.4)	20 (2.4)
	1–14	81 (9.6)	60 (7.1)	21 (2.5)
	15–24	69 (8.1)	61 (7.2)	8 (0.9)
	25–44	331 (39.2)	288 (34.1)	43 (5.09)
	45–64	193 (22.9)	153 (18.1)	40 (4.7)
	> 65	130 (15.4)	106 (12.6)	24 (2.8)
	Total	844 (100)	688 (81.5)	156 (18.5)

### Distribution of bacterial pathogens

The spectrum of bloodborne bacteria in the present study is shown in Table 2. A total of 156 bacterial isolates were documented, of which 79 (50.6%) were Gram-negative and 77 (49.4%) were Gram-positive. *K. pneumoniae* was the dominant Gram-negative bacterium (14.1%), followed by *E. coli* (9.0%) and *Acinetobacter lwoffii* (5.8%)*. Enterobacter cloacae, P. aeruginosa, and Acinetobacter baumannii* were recovered in the same proportion, i.e., 4.5%. *Staphylococcus epidermidis,* a coagulase-negative *Staphylococcus* species, was the dominant Gram-positive isolate (17; 10.9%) followed by *S. aureus* (15; 9.6%). *Enterococcus faecalis* was the only non-*Staphylococcus* Gram-positive bacterium isolated in our study.

**Table 2:** Percentage distribution of blood-born bacteria among the total isolates (n = 156).

Category	Species	Number of isolates and % total isolates (n, %)
Gram-negative	*Klebsiella pneumoniae*	22 (14.1)
	*Klebsiella oxytoca*	4 (2.6)
	*Acinetobacter baumannii*	7 (4.5)
	*Acenitobacter lwoffii*	9 (5.8)
	Achromobacter xylosoxidan	2 (1.3)
	*Escherichia coli*	14 (9.0)
	*Enterobacter cloacae*	7 (4.5)
	*Pseudomonas aeruginosa*	7 (4.5)
	*Moraxella catarrhalis*	3 (1.9)
	*Salmonella typhi*	1 (0.6)
	*Pantoea septica*	1 (0.6)
	*Leclercia. Adecarboxylata*	1 (0.6)
	*Serratia marcescens*	1 (0.6)
	**Gram-negative total**	**79 (50.6)**
Gram-positive	*Staphylococcus aureus*	15 (9.6)
	*Enterococcus faecalis*	6 (3.8)
	*Staphylococcus. Lugdunesis*	3 (1.9)
	*Staphylococcus warneri*	12 (7.8)
	*Staphylococcus epidermidis*	17 (10.9)
	*Staphylococcus intermedius*	5 (3.2)
	*Staphylococcus haemoliyticus*	9 (5.8)
	*Staphylococcus hominis*	6 (3.8)
	*Staphylococcus saprophyticus*	1(0.6)
	*Staphylococcus xylosus*	3 (1.9)
	**Gram-positive total**	**77 (49.4)**

Significant values are in bold.

### Percentage antimicrobial resistance profile of Gram-negative bacteria

Table 3 shows the overall percentage antimicrobial resistance profile of Gram-negative bacteria as well as the drug resistance profile of each pathogen per drug category. The overall drug resistance profile was the highest against ampicillin (80.8%) and the lowest against imipenem (5.2%). The drug resistance profile of the most commonly isolated Gram-negative bacterial pathogen was as follows: Resistance to Carbapenem: *K. pneumoniae* with a resistance rate of 2.0% to meropenem versus 1.0% to imipenem; *E. coli* with a resistance rate of 1.0% to imipenem versus 7.1% to meropenem; *A. baumannii* with a resistance rate of 0% to both meropenem and imipenem; *E. cloacae* with a resistance rate of 0% to both meropenem and imipenem, and *P. aeruginosa* with a resistance rate of 14.3% to meropenem versus 1.0% to imipenem. Resistance to cephalosporins:—Gram-negative bacteria were tested against 9 cephalosporin drugs. Out of 22 isolates of *K. pneumoniae* tested ≥ 82% of the isolates were resistant to 8 cephalosporin drugs except for cefoxitin with a resistance rate of 9.1%. Of the 14 isolates *of E. coli* tested, ≤ 50% of the isolates were resistant to 7 drugs, but 71.4% to cephalothin and 57.1% to cefazolin. The resistance rate of *A. baumannii* to cephalosporin drugs extended from 71.4% to 100%, where the isolates were 100% resistant to six cephalosporin drugs. The resistance rate of *P. aeruginosa* was 100% to 7 cephalosporin drugs except for cefepime (28.6%) and ceftazidime (42.9%). Resistance rate to aminoglycosides: *K. pneumoniae*, 32% to tobramycin vs 72.7% to gentamycin; *E. coli,* 14.3% to tobramycin vs 28.6% to gentamycin; *A. baumannii,* 28.6% to both tobramycin and gentamycin; *P. aeruginosa,* 14.3% to both tobramycin and gentamycin. Resistance rate against fluoroquinolones:—*K. pneumoniae*, 13.6% to levofloxacin vs 40.9% to ciprofloxacin; *E. coli* 50% to both levofloxacin and ciprofloxacin; *A. baumannii* 26.6% to levofloxacin vs 71.4% to ciprofloxacin; *P. aeruginosa,* 26.6 to levofloxacin vs 30% to ciprofloxacin. Resistance to β -lactam/β-lactamase inhibitor combination drugs:—*K. pneumoniae* 50.0% to ampicillin/clavulanic acid vs 32% to piperacillin/ tazobactam), *E. coli, 21.4% to ampicillin*/ clavulanic acid *vs 14.3%* piperacillin/tazobactam; *A. baumannii 100% to* ampicillin/ clavulanic acid vs 71.4% to piperacillin/ tazobactam; *P. aeruginosa,100% to* ampicillin/clavulanic acid vs 42.9% to piperacillin/ tazobactam.

**Table 3:** Percentage antimicrobial resistance profile of blood-born Gram-negative bacteria.

Species & number (n)		Antimicrobial drugs tested, n (%)																				
		AMP	AMC	TZP	CFA	CFZ	CFU	CFA	FOX	CPD	CAZ	CRO	CFP	GM	TBM	CIP	LEV	TEC	NFT	SXT	MFP	IMP
*K*. *pneumoniae* (22)	n	22	11	7	20	19	19	19	2	18	18	18	18	16	7	9	3	14	7	16	2	1
	%	100	50	32	91	86	86	86	9.1	82	82	82	82	72.7	32	40.9	13.6	63	31.8	72.7	9	4.5
*K*. *oxytoca* (4)	n	4	2	1	3	3	3	3	3	3	3	3	3	3	3	1	1	3	1	3	0	0
	%	100	50	25	75	75	75	75	25	75	75	75	75	75	75	25	25	75	25	75	0	0
*A. baumannii* (7)	n	7	7	5	7	7	7	7	7	7	5	5	5	2	2	5	2	1	6	5	1	1
	%	100	100	71.4	100	100	100	100	100	100	71.4	71.4	71.4	28.6	28.6	71.4	28.6	14.3	85.7	71.4	14.3	14.3
*A*. *lwoffii* (9)	n	0	0	0	5	4	0	0	4	0	0	0	0	0	0	0	0	0	0	0	0	0
	%	0	0	0	55.5	44.4	0	0	44.4	0	0	0	0	0	0	0	0	0	0	0	0	0
*A*. *xylosoxidan* (2)	n	–	–	–	–	–	–	–	–	–	–	–	–	–	–	–	–	–	–	–	–	–
	%	–	–	–	–	–	–	–	–	–	–	–	–	–	–	–	–	–	–	–	–	–
*E. coli* (14)	n	12	3	2	10	8	7	7	2	7	7	7	6	4	2	7	7	10	1	10	1	0
	%	85	21.4	14.3	71.4	57.1	50.0	50.0	14.3	50.0	50.0	50.0	42.9	28.6	14.3	50.0	50.0	71.4	7.1	71.4	7.1	0
*E*. *cloacae* (7)	n	7	7	0	7	7	4	7	7	7	0	3	0	4	0	0	0	3	3	4	0	0
	%	100	100	0	100	100	57.1	100	100	100	0	42.9	0	57.1	0	0	0	42.9	42.9	57.1	0	0
*P. aeruginosa* (7)	n	7	7	3	7	7	7	7	7	7	3	7	2	1	1	2	2	7	7	7	2	1
	%	100	100	42.9	100	100	100	100	100	100	42.9	100	28.6	14.3	14.3	30	28.6	100	100	100	28.6	14.3
*M. catarrhalis* (3)	n	0	0	3	3	3	3	0	0	0	0	3	0	0	0	0	0	0	0	0	0	0
	%	0	0	100	100	100	100	0	0	0	0	100	0	0	0	0	0	50	0	0	0	0
*S. Typhi* (1)	n	1	1	0	1	1	1	1	1	0	0	0	0	0	0	0	0	0	0	0	0	0
	%	100	100	0	100	100	100	100	100	0	0	0	0	0	0	0	0	0	0	0	0	0
*P*. *septica* (1)	n	1	1	0	0	0	0	0	0	0	0	0	0	0	0	0	0	0	0	0	0	0
	%	100	100	0	0	0	0	0	0	0	0	0	0	0	0	0	0	0	0	0	0	0
*L*. *adecarboxylata* (1*)*	n	0	0	0	0	0	0	0	0	0	0	0	0	0	0	0	0	0	0	0	0	0
	%	0	0	0	0	0	0	0	0	0	0	0	0	0	0	0	0	0	0	0	0	0
*S. marcescens* (1)	N	1	1	1	1	1	1	1	0	0	0	0	0	0	0	0	0	0	0	0	0	0
	%	100	100	100	100	100	100	100	0	0	0	0	0	0	0	0	0	0	0	0	0	0
Total isolates = 77	n	62	40	22	64	60	52	52	33		36	46	34	30	15	22	15	38	25	45	5	4
	%	80.8	51.9	28.6	70.1	77.9	67.5	67.5	42.6		46.6	59.7	44.2	38.9	19.5	28.6	19.5	49.4	32.5	58.4	6.5	5.2

*AMP* ampicillin, *AMC* amoxicillin/clavulanic acid, *TZP* piperacillin/tazobactam, *CFA* cephalothin, *CFZ* cefazoline, *CFU* cefuroxime, *CFXA* cefuroxime-Axetile, *FOX* cefoxitin, *CPD* cefpodoxime, *CAZ* ceftazidime, *CRO* ceftriaxone, *CFP* cefepime, *GM* gentamicin, *TBM* tobramycin, *CIP* ciprofloxacin, *LEV* levofloxacin, *TEC* tetracycline, *NFT* nitrofurantoin, *SXT* trimethoprim/sulfamethoxazole and *MEM* meropenem *DT* drugs tested (n, %), – antibacterial drug profile was not reported by the machine.

### Percentage antimicrobial resistance profile of Gram-positive bacteria

The antimicrobial resistance profile of Gram-positive cocci is shown in Table 4. Gram-positive bacteria were more resistant to clindamycin (70.1%), erythromycin (63.6%) tetracycline (61.0%). The resistance rate of Gram-positive bacteria against linezolid (2.6%), nitrofurantoin (2.6%), moxifloxacin (6.5%), and gentamycin (9.1%) was very low. Vancomycin (glycopeptide) and daptomycin (a cyclic lipopeptide antibiotic) showed a 100% susceptibility to all Gram-positive bacteria. *E. faecalis* showed a high level of resistance toward erythromycin (83.3%), minocycline (83.3%), tetracycline (83.3%), and quinupristin/dalfopristin (100%) while the species was 100% susceptible to high-level antibiotics such as vancomycin, daptomycin, and linezolid, a synthetic drug of the class oxazolidinones.

**Table 4:** Percentage antimicrobial resistance profile of blood-born Gram-positive bacteria.

Species & number		Antimicrobial drugs tested, n (%)															
		CIP	CM	E	GM	LEV	MNO	MXF	FT	QDA	RA	TE	TGC	SXT	LIM	VA	DAP
*Staphylococcus aureus (15)*	n	5	10	10	3	3	2	0	0	3	5	10	5	8	0	0	0
	%	33.3	66.7	66.7	20.0	20	13.3	0	0	20.0	33.3	66.7	33.3	53.3	0	0	0
*Enterococcus faecalis (6)*	n	1	1	5	0	0	5	0	0	6	0	5	1	1	0	0	0
	%	16.7	16.7	83.3	0	0	83.3	0	0	100	0	83.3	16.7	16.7	0	0	0
*Staphylococcus Lugdunesis (3)*	n	0	1	3	0	3	0	3	1	0	0	3	0	0	0	0	0
	%	0	33.3	100	0	100	0	100	33.3	0	0	100	0	0	0	0	0
*Staphylococcus warneri (12)*	n	5	8	9	0	3	3	1	1	1	5	9	0	7	0	0	0
	%	41.7	66.7	75.0	0	25.0	25.5	8.3	8.3	8.3	41.7	75.0	0.0	58.3	0	0	–
*Staphylococcus epidermidis (17)*	n	10	17	13	0	10	2	0	0	8	9	10	4	4	1	0	0
	%	58.8	100	76.5	0	58.8	11.8	0	0	47.1	52.9	58.8	23.5	23.52	5.9	0	0
*Staphylococcus intermedius (5)*	n	1	4	1	3	3	1	0	0	1	3	3	1	1	1	0	0
	%	20.0	80.0	20.0	60.0	60.0	20.0	0	0	20.0	60.0	60.0	20.0	20.0	20.0	0	0
*Staphylococcus haemoliyticus (9)*	n	0	5	0	0	0	0	0	0	0	5	0	0	0	0	0	0
	%	0	55.5	0	0	0	0	0	0	0	55.6	0	0	0	0	0	0
*Staphylococcus hominins (6)*	n	4	6	6	0	4	3	0	0	4	4	5	0	3	0	0	0
	%	66.7	100	100	0	66.7	50	0	0	66.7	66.7	83.3	0	50	0	0	0
*Staphylococcus saprophyticus (1)*	n	0	1	0	0	0	0	0	0	0	1	0	0	0	0	0	0
	%	0	100	0	0	0	0	0	0	0	100	0	0	0	0	0	0
*Staphylococcus xylosus (3)*	n	1	1	2	1	1	0	1	0	1	2	2	0	2	0	0	0
	%	33.3	33.3	66.7	33.3	33.3	0	33.3	0	33.3	66.7	66.7	0	66.7	0	0	0
Total 77	n	27	54	49	7	27	16	5	2	24	34	47	11	26	2	0	0
	%	35.1	70.1	63.6	9.1	35.1	20.8	6.5	2.6	31.2	44.1	61.0	14.3	33.8	2.6	0	0

*CIP* ciprofloxacin, *CM* clindamycin, *E* erythromycin, *GM* gentamicin, *LEV* levofloxacin, *MN0* minocycline, *MXF* moxifloxacin, *FT* nitrofurantoin, *QDA* quinupristin/dalfopristin, RA rifampicin, *TE* tetracycline, *TGC* tigecycline, *LIN* linezolid, *SXT* trimethoprim/sulfamethoxazole, *VA* vancomycin, *DAP* daptomycin, *DT* drugs tested (n, %).

### Multi-drug-resistant profile of Gram-negative bacteria

The MDR pattern of Gram-negative bacteria was determined by considering the following nine classes of antibiotics: Penicillin, cephalosporin; aminoglycosides; quinolones (fluoroquinolones); trimethoprim/sulfamethoxazole; tetracyclines; nitrofurantoin, combination drugs, and carbapenems. The overall prevalence of MDR Gram-negative bacteria was 41.6 (32/79) of which 77.3% (17/22) of *K. pneumoniae*, 85.8% (6/7) of *A. baumannii, 28.6*% (2/7) of *P. aeruginosa*, and 28.6% (4/14) of *E. coli* were MDR (Table 5).

**Table 5:** The multi-drug resistance level of blood-born Gram-negative bacteria.

Isolates (number)	Level of antibiotic resistance n (%)								Total isolates (≥ R3)
	R0	R1	R2	R3	R4	R5	R6	≥ R7	
*K*. *pneumoniae* (22)	0 (0)	1 (4.5)	1 (4.5)	3 (13.6)	3 (13.6)	4 (18.2)	6 (27.3)	1 (4.5)	17 (77.3%)
*K*. *oxytoca* (4)	0 (0)	2 (50.0)	1 (25.5)	1 (25.)	0 (0)	0 (0)	0 (0)	0 (0)	1 (25.0%)
*A. baumannii* (7)	0 (0)	0 (0)	1 (14.3)	1 (14.3)	1 (14.3)	1 (14.3)	1 (14.3)	2 (28.6)	6 (85.8%)
*A*. *lwoffii* (9)	2 (22.2)	2 (22.2)	4 (44.4)	1 (11.1)	0 (0)	0 (0)	0 (0)	0 (0)	1 (11.1%)
*E. coli* (14)	1 (7.1)	5 (35.7)	4 (28.56)	2 (14.3)	2 (14.3)	0 (0)	0 (0)	(0)	4 (28.6%)
*E*. *cloacae* (7)	2 (28.6)	2 (28.6)	2 (28.6)	1 (14.3)	(0)	(0)	(0)	(0)	1 (14.3%)
*P. aeruginosa* (7)	0 (0)	1 (14.3)	2 (28.6)	1 (14.3)	1 (14.3)	1 (14.3)	1 (14.3)	(0)	2 (28.6%)
*M. catarrhalis* (3)	0 (0)	1 (33.3)	2 (66.6)	0 (0)	0 (0)	0 (0)	0 (0)	0 (0)	0 (0%)
*S. typhi* (1)	0 (0)	0 (0)	1 (100)	0 (0)	0 (0)	0 (0)	0 (0)	0 (0)	0 (0%)
*P*. *septica* (1)	0 (0)	0 (0)	1 (100)	0 (0)	0 (0)	0 (0)	0 (0)	0 (0)	0 (0%)
*L*. *adecarboxylata* (1)	0 (0)	1 (100)	0 (0)	0 (0)	0 (0)	0 (0)	0 (0)	0 (0)	0 (0%)
*A*. *xylosoxidan* (2)	0 (0)	1 (50.0)	1 (50.0)	0 (0)	0 (0)	0 (0)	0 (0)	0 (0)	0 (0%)
*S. marcescens* (1)	0 (0)	1 (100)	0 (0)	0 (0)	0 (0)	0 (0)	0 (0)	0 (0)	0 (0%)
*Total 77*	5 (6.5)	17 (22.1)	20 (26.0)	11 (14.3)	7 (9.1)	6 (7.8)	8 (10.4)	3 (3.9)	32 (41.6)

*R0* resistance to no antibiotics, *R1* resistance to one antimicrobial category, *R2* resistance to two antimicrobial categories, *R3* resistance to three antimicrobial categories, *R4* resistance to four antimicrobial categories, *R5* resistance to five antimicrobial categories, *R6* resistance to six antimicrobial categories, *resistance* ≥ *7* resistance to seven antimicrobial categories, ≥ *R3* nonsusceptibility to at least one agent in 3 or more classes of antibiotics.

### Multidrug-resistant profile of Gram-positive bacteria

The MDR pattern of Gram-positive bacteria was determined by considering the following classes of antibiotics: aminoglycosides; quinolones (fluoroquinolones); trimethoprim/sulfamethoxazole; tetracyclines; nitrofurantoin, glycopeptide; a cyclic lipopeptide antibiotic; oxazolidinones; glycylcycline; streptomycin; lincomycin; macrolide; antimicrobials. The overall MDR prevalence rate of Gram-positive bacteria was 58.2 (46/77) of which 66.7% (10/15) of *S. aureus*, 66.7% (4/6) of *E. faecalis*, 66.7% (8/12) of *S. warneri*, and 52.9% (9/17) of S. epidermidis were MDR (Table 6).

**Table 6:** The multi-drug resistance level of blood-born Gram-positive bacteria.

Isolates (number)	Level of antibiotic resistance n (%)								Total isolates (≥ R3)
	R0	R1	R2	R3	R4	R5	R6	≥ R7	
*S. aureus (15)*	0 (0)	2 (13.3)	3 (20.0)	2 (13.3)	4 (26.6)	2 (13.3)	2 (13.3)	0 (0)	10 (66.7)
*E. faecalis (6)*	0 (0)	1 (16.7)	1 (16.7)	1 (16.7)	2 (33.3)	1 (16.7)	0 (0)	0 (0)	4 (66.7)
*S. Lugdunesis (3)*	0 (0)	1 (33.3)	0 (0)	1 (33.3)	1 (33.3)	0 (0)	0 (0)	0 (0)	1 (33.3)
*S. warneri (12)*	1 (8.3)	2 (16.7)	1 (8.3)	2(16.7)	3 (25.0)	1 (8.3)	1 (8.3)	1 (8.3)	8 (66.7)
*S. epidermidis (17)*	1 (5.9)	4 (23.5)	3 (17.6)	2 (11.8)	2 (11.8)	3 (17.6)	1 (5.9)	1 (5.9)	9 (52.9)
*S. intermedius (5)*	0 (0)	2 (40.0)	1 (20.0)	2 (40.0)	1 (20.0	0 (0)	0 (0)	0 (0)	3 (60.0)
*S. haemoliyticus (9)*	1 (11.1)	1 (11.1)	2 (22.2)	1 (11.1)	2 (22.2)	1 (11.1)	1 (11.1)	0 (0)	5 (55.6)
*S. hominis (6)*	0 (0)	2 (33.3)	1 (16.7)	1 (16.7)	1 (16.7)	1 (16.7)	0 (0)	0 (0)	3 (50.0)
*S. saprophyticus (1)*	0 (0)	0 (0)	0 (0)	0 (0)	1 (100)	0 (0)	0 (0)	0 (0)	1 (100)
*S. xylosus (3)*	0 (0)	0 (0)	1 (33.3)	1 (33.3)	1 (33.3)	0 (0)	0 (0)	0 (0)	2 (66.7)
Total 77	3 (3.9)	15 (19.5)	13 (16.9)	13 (16.9)	18 (23.4)	9 (11.7)	5 (6.5)	2 (2.6)	46 (59.7)

*R0* resistance to no antibiotics, *R1* resistance to one antimicrobial category, *R2* resistance to two antimicrobial categories, *R3* resistance to three antimicrobial categories; *R4*, resistance to four antimicrobial categories *R5* resistance to five antimicrobial categories, *R6* resistance to six antimicrobial categories, *resistance* ≥ *7* resistance to seven antimicrobial categories, ≥ *R3* nonsusceptibility to at least one agent in 3 or more classes of antibiotics.

### Prevalence of extended-spectrum β- lactamase (ESBL) producing gram-negative bacteria

The overall prevalence of ESBL-producing Gram-negative was 26 (34.2%). There was intra-species variation in ESBLs production in which the highest percentage was recorded among *K. pneumoniae*, 55.5% (12/22) followed by *E. coli*, 50.0% (6/14), and the lowest production observed in *K. oxytoca* with 25% 1/4) (Table 7).

**Table 7:** The magnitude of ESBLs Production in Gram-negative bacilli.

Species	Frequency	Percentage
*K*. *pneumoniae* (22)	12	55.0
*K*.* oxytoca* (4)	1	25.0
*A. baumannii* (7)	3	42.9
*E*. *cloacae* (7)	2	28.6
*E. coli* (14)	6	50.0
*P. aeruginosa* (7)	2	28.6
*Total*	26	32.9

## Discussion

In the present study, out of 844 blood samples processed for culture, 156 (18.5%) were culture-positive. Our finding was lower than BSI reported by earlier studies^15–18^ but higher than other studies^19–21^. The disparity in blood culture positivity rates in different studies could be attributed to differences in result interpretation, the volume of blood used (5 ml versus 10 ml), sample size, and the number of blood cultures to which the blood sample was inoculated (one versus two blood culture bottles).

In our study, 50.6% of BSIs were caused by Gram-negative while 49.4% were by Gram-positive bacteria. Gram-positive predominance BSIs (54% Gram-positive vs 45% Gram-negative) by Arega et al.^16^, (88.8% Gram-positive vs 11.2% Gram-negative) by Sharma et al.^17^, (52.7% Gram-positive vs 47.3% Gram-negative) by Arora et al.^18^, and (82.1% Gram-positive vs 17.9% Gram-negative bacteria) by Moyo et al.^19^ were documented. On the other hand, Gram-negative bacteria's predominance in causing BSIs was reported by many other researchers^15,20^. In the current work, coagulase-negative staphylococci Gram-positive bacteria, were the most common bacterial pathogen causing BSI. Our result was in concordance with various studies^12,21^. Although about > 85% of CoNS was noted as contaminants in the past^22^, studies have shown that CoNS are frequently associated with pediatric bloodstream infections and adults in settings where implanting of intravascular catheters and indwelling prosthetic devices are practiced^23,24^. The high prevalence of CoNS in the present study could partly be explained by the fact that about 19.0% of blood was collected from patients aged below 15 years and/or may be due to the use of an improved and highly sensitive automated machine for bacterial identification. However, as CoNS are also possible skin contaminants, their pathogenic status should be verified by other means before starting therapy. *Staphylococcus aureus* was the second most frequent cause of BSI followed by *E. faecalis. S. aureus* as the commonest Gram-positive bacterium causing BSIs has been demonstrated by other studies^23,24^.

Among the many Gram-negative bacteria reported in our study, those identified as serious life-threatening pathogens by WHO^7^ such as *K. pneumoniae, A. baumannii, P. aeruginosa*, and *Enterobacter spp* were the commonest isolates. Our result concurs with the report of Tsegaye et al.^15^ Khan et al.^21^, Kasanga et al.^25^ Ahmed et al.^26^.

Evaluation of the drug resistance pattern of Gram-negative bacterial pathogens to different drug categories demonstrated that they were highly resistant to most drug categories except carbapenems. Their percentage drug resistance rate extended from 5.2% for imipenem to 80.8% for ampicillin. Their overall drug resistance profile to the nine cephalosporin drugs tested, except for cefoxitin, ceftazidime, and cefepime was very high. Importantly, cephalosporins were less effective against the most common and important pathogens. *Klebsiella pneumoniae* with a resistance rate extending from 82.0% to 91.0% except for cefoxitin (9.1%); *A. baumannii* with a resistance rate extending from 71.4% to 100%; *P. aeruginosa* with a resistance rate of 100% except for ceftazidime (42.9%) and cefepime (28.6%) was alarming. Our finding was similar to that of Hautala et al.^27^ that demonstrated the frequency of drug-resistant Gram-negative bacteria to cephalosporins varied from 75.0% for cefazolin to 84.2% for cefuroxime. Our result also agreed marginally with the results of recent studies conducted in Ethiopia^28–32^. Such high levels of drug resistance by Gram-negative bacteria to cephalosporin could be attributed that they have been used in many settings for empirical treatment mainly due to their broad-spectrum activity and low toxicity. More importantly, however, Gram-negative bacteria are the main producers of enzymes (ESBLs) that inactivate drugs with beta-lactam functional groups such as cephalosporins. The observation of a high drug-resistant profile of Gram-negative bacteria to cephalosporines was suggestive of the fact that 50.0% *E. coli*, 55.0% *K. pneumoniae*, 42% of *A. baumannii*, and 28.6% *P. aeruginosa* isolates in the present study were ESBL producers. Why *E. coli* was comparatively less resistant to cephalosporins in the present work given that the bacterium was the second most producer of ESBL in the current study was obscure. Similarly, less resistance rate of *E. coli* to cephalosporins was reported from Nepal^33^. Therefore, with caution, our study demonstrated that similar to penicillins, new cephalosporins (2nd–4th generations) synthesized with an expansion of their activities against Gram-negative rods as the goal is declining. Gram-negative bacteria isolates were highly sensitive to carbapenem with a resistance level of 6.5% to meropenem vs 5.2% to imipenem. A lower resistance rate of Gram-negative bacteria to carbapenem (1.7%) to both meropenem and imipenem than our study has been reported in a study conducted in Ethiopia by Beyene et al.^28^. However, higher resistance rates of Gram-negative bacteria to carbapenem than our study have been reported in related studies conducted in Ethiopia^29,31,32^. Despite the poor drug-controlling system in Ethiopia coupled with the absence of drug stewardship, the resistance of Gram-negative bacteria to carbapenem is generally low. The high sensitivity of Gram-negative bacteria to carbapenems could be attributed to the fact that carbapenems are highly controlled antibiotics (they are not readily available over the counter) in Ethiopia. Furthermore, carbapenems are β-lactam drugs that are structurally different from penicillins and cephalosporins that are not easily inactivated by most β-lactamases. Yet the prevalence of drug resistance to the carbapenems in our study is frightening in a country where alternative antibiotics are scarce. Moreover, the emergence and spread of carbapenem-resistant bacteria are more problematic in developing countries due to the lack of laboratory capacity for their diagnosis.

The analysis of the drug resistance profile of the most commonly isolated Gram-negative bacteria per drug category demonstrated variable results. The resistance rate of *K. pneumoniae*, the commonest Gram-negative bacterium in our study was 9.0% to meropenem and 4.5% to imipenem. Our result agreed with the results of studies conducted in Ethiopia^31^, and China^34^. The occurrence of resistant isolates of *K. pneumoniae* to aminoglycoside in the present work was variable being 72.7% for gentamycin and 32.0% for tobramycin. Similar results were reported from Ethiopia by many studies^15,28,30,32^. Yet a seventy percent resistance rate of the pathogen to tobramycin reported by Beyene et al.^28^ was extremely high compared to our result. The incidence of resistant isolates of the pathogen to quinolones in our study was 13.6% for levofloxacin and 41.0% for ciprofloxacin which is lower than compared resistance rates that extend from 40.0% to 69.0%^15,28,30,32^. Among drugs of β-lactam/β-lactamase inhibitor combinations, piperacillin/tazobactam was better active against *K. pneumoniae* with a resistance rate of 26.6% than amoxicillin/ clavulanic acid with a resistance level of 52.0%. A notable difference was not observed between our result and the results reported by studies conducted in Ethiopia^28,29^.

The resistance rate of *E. coli* to carbapenem in our study was 7.1%; (0.0% to imipenem vs 7.1% to meropenem). Our finding was similar to reports from China^34^, USA^35^, and Europe^36,37^. A resistance rate of 28.8% to both gentamycin and tobramycin of the pathogen in our study was comparable with 5–25% from Europe^36,37^. A 50% resistance of *E. coli* to both levofloxacin and ciprofloxacin in this study correlated with quinolone resistance of *E. coli* in the USA (41.8%) and EU countries (11 to 52%)^38^. The pathogen was susceptible to β-lactam/β-lactamase inhibitor combinations, piperacillin/tazobactam with a resistance rate of 14.3%, and amoxicillin/ clavulanic acid with a resistance rate of 21.4%.

Over 70% of *A. baumannii* was resistant to 15 drugs of which 100% of the isolates were resistant to eight drugs. Furthermore, 14.3% of the pathogen was resistant to carbapenems, the most active drugs to Gram-negative bacteria in our study demonstrating that *Acenitobacter* species were the most carbapenem-resistant next to *P. aeruginosa*. It has been shown that > 50% of isolates of *A. baumannii* were resistant to carbapenems, quinolones, and aminoglycosides in Europe^38^.

*Pseudomonas aeruginosa* in our study was also 100% resistant to 12 drugs and the resistance rate of the bacterium was 28.0% to meropenem and 14.3% to imipenem. The resistance rate of the bacterium to aminoglycosides is 14.3% to both gentamycin and tobramycin, quinolones (0% to ciprofloxacin vs 28.6% to levofloxacin), and cefepime (28.6%) are relatively low. In Europe, *P. aeruginosa* with high resistance rates to aminoglycosides, ceftazidime, quinolones, piperacillin-tazobactam, and carbapenems have been reported^39^.

The higher drug resistance of the non-fermentative Gram-negative bacteria compared to the enterobacteria noted in our study is not amazing. Non-fermentative Gram-negative bacteria are recognized to be naturally resistant to the most important classes of antibiotics. Higher intrinsic resistance in these bacteria has been associated with their lower cellular permeability and higher efflux activities^39^. It has been noted that the drug resistance rate against Gram-negative bacteria was higher for meropenem than imipenem, ciprofloxacin than levofloxacin, gentamycin than tobramycin and amoxicillin/clavulanic acid than piperacillin-tazobactam. The pattern of drug resistance noted in our work was associated with the pattern of antibiotic use in Ethiopia. Generally, meropenem, ciprofloxacin, gentamycin and amoxicillin/clavulanic acid are the most commonly prescribed antibiotics in Ethiopia. Higher resistance rates of these drugs than drugs in the same drug category supported the notion that extensive use of antibiotics is a key driving force for the development of drug resistance^40^.

The frequency of drug resistance in Gram-positive cocci differed from 0.0% for vancomycin and daptomycin to 70.1% for clindamycin. Their drug resistance rate to the commonly prescribed antibiotics was high and corresponded with the findings of related studies in the country^15,18^. The high antibiotic resistance reported against these drugs in this study may be due to easy accessibility over the counter to most antibiotics and a high selection pressure due to the extensive use of these antibiotics. However, Gram-positive bacteria showed 100% susceptibility to vancomycin and daptomycin, although reduced susceptibility to glycopeptides (vancomycin) in *S. aureus* has emerged during the last decades. All Gram-positive bacterial isolates were also 100% susceptible to daptomycin despite the development of resistance to this lipopeptide antibiotic reported by Chong et al.^41^. All isolates of Gram-positive bacteria in our study were also 100% susceptible to linezolid except *S. epidermidis* with a resistance rate of 5.9% and *S. intermedius* with a resistance rate of 20%. About 1–5% linezolid-resistant coagulase-negative staphylococci have been reported to oxazolidinone by Decousser et al.^42^. *Enterococcus faecalis* was extremely resistant to erythromycin (83.3%), minocycline (83.3%), quinupristin (100%) tetracycline (83.3%) nevertheless all strains of the bacterium were 100 susceptible to vancomycin, daptomycin, and linezolid. Glycopeptide resistance in enterococci is a serious problem in the USA. It has been reported that by 2007, > 80% of *E. faecium* isolates in USA hospitals were resistant to vancomycin^42,43^.

The phenotypic data generated in the current study showed that 32.9% of Gram-negative bacteria were ESBL producers. The prevalence of ESBL production noted in the present study did not substantially deviate from earlier studies conducted within and outside Ethiopia^31,44–46^. Nevertheless, the higher prevalence rate of ESBL production than the prevalence of the current study was reported within Ethiopia and abroad^29,30,44–46^. *K. pneumoniae* (55.0%), *E. coli* (50.0%), and *A. baumannii* (42.9%) were the commonest ESBL producers in the present study. *E. coli* with a frequency of 83.13% and *K. pneumoniae* with a frequency of 78.84% are reported as the two major ESBL producers in Mexico^46^. Similarly, *E. coli* with a frequency of 70.9% and *Klebsiella* spp. with a magnitude of 59.4% are recorded as the commonest ESBL produces in Nepal^33^.

The rapid increase and spread of multidrug-resistant bacteria are major threats to public health all over the world. The problem is more significant in Enterobacteriaceae because of their omnipresence in the environment and the relative ease of gaining plasmids containing genes that encode for ESBLs and other resistance genes that confer resistance to many other classes of antibiotics^47^. The magnitude of MDR bacteria in this study was 41.6% which is lower than almost two-fold prevalence rates reported by, Moges et al. (85.8%)^30^ Beyene et a, (94.5%)^28^, Alebel et al. (81.1%)^32^ nevertheless comparable with prevalence rate demonstrated from studies conducted in Ethiopia by Abdeta et al. 45.2%^29^ Perez^48^, Bitew et al. (41.2%)^49^, Biset et al. 56.7%^44^. In the current study, 85% of *A. baumannii*, 77.3% of *K. pneumoniae,* (28.6%), *of E. coli*, and (28.6%) *of P. aeruginosa* were multidrug-resistance strains. About 32 multidrug-resistant bacteria out of a total of 79 Gram-negative bacteria were high. As a developing country, increased use of drugs over the counter, incomplete courses of therapy, and prolonged therapy for recurrent bacterial diseases are commonly practiced in Ethiopia. These practices could be cited as possible factors for the high prevalence of MDR Gram-negative bacterial species noted in the current study.

The overall prevalence of MDR Gram-positive bacteria in our study was 59.7%. About 66.7% of *S. aureus,* 66.7% *of E. faecalis*, 66.7% of *S. warneri*, and 52.9% of *S. epidermidis* were MDR. The overall prevalence of MDR Gram-positive bacteria in our study was higher than those reported by Asres et al. (53.3%)^50^, and Azene et al. (52.7%)^51^ but lower than those reported by Godebo et al. (66%)^52^ and Alam et al. (69.0%)^12^. The possible explanation for such irregularities in the prevalence of MDR bacteria might be the difference in study settings where previous studies solely included inpatients where higher MDR strains are expected. *S. aureus* was the most predominant MDR bacterium in almost all of these studies.

## Limitations of the study

The drug resistance reported in this study is primarily phenotypic. To this end determination of drug resistance genes by molecular methods is the aim of our future studies.

## Conclusion

The prevalence of bloodstream infection was 18.5%. Yet most bacterial species isolated in the study were serious life-threatening bloodborne pathogens. Multidrug resistance in blood-borne pathogens and ESBL production in Gram-negative bacteria were high. Although carbapenem resistance prevalence was low, the recognized resistance to the drugs in our study is alarming since alternative drugs and laboratory capacity for their detection are hardly available. All these situations call for the establishment of strong infection control strategies, a drug regulatory system, and established antibiotic stewardship in healthcare settings.

## Data Availability

The dataset generated and/or analyzed during the current study is available from the corresponding author upon reasonable request.
